# Engineering Vehicles Detection Based on Modified Faster R-CNN for Power Grid Surveillance

**DOI:** 10.3390/s18072258

**Published:** 2018-07-13

**Authors:** Xuezhi Xiang, Ning Lv, Xinli Guo, Shuai Wang, Abdulmotaleb El Saddik

**Affiliations:** 1 College of Information and Communication Engineering, Harbin Engineering University, Harbin 150001, China; lvning@hrbeu.edu.cn (N.L.); guoxinli@hrbeu.edu.cn (X.G.); wangshuai_0121@163.com (S.W.); 2 School of Electrical Engineering and Computer Science, University of Ottawa, Ottawa, ON KIN 6N5, Canada; elsaddik@uottawa.ca

**Keywords:** power grid surveillance, external damage, engineering vehicles, faster R-CNN, transfer learning

## Abstract

Engineering vehicles intrusion detection is a key problem for the security of power grid operation, which can warn of the regional invasion and prevent external damage from architectural construction. In this paper, we propose an intelligent surveillance method based on the framework of Faster R-CNN for locating and identifying the invading engineering vehicles. In our detection task, the type of the objects is varied and the monitoring scene is large and complex. In order to solve these challenging problems, we modify the network structure of the object detection model by adjusting the position of the ROI pooling layer. The convolutional layer is added to the feature classification part to improve the accuracy of the detection model. We verify that increasing the depth of the feature classification part is effective for detecting engineering vehicles in realistic transmission lines corridors. We also collect plenty of scene images taken from the monitor site and label the objects to create a fine-tuned dataset. We train the modified deep detection model based on the technology of transfer learning and conduct training and test on the newly labeled dataset. Experimental results show that the proposed intelligent surveillance method can detect engineering vehicles with high accuracy and a low false alarm rate, which can be used for the early warning of power grid surveillance.

## 1. Introduction

Stable and reliable operation of the power system is the fundamental guarantee for the development of power supply security. As an important part of the power supply system, the security of overhead high-voltage transmission lines is closely related to the normal operation of the entire power system. Since the power supply distance is so long, the overhead high-voltage transmission line frequently passes through cities and suburbs, where construction sites are often presented. In recent years, with the development of the construction of urban infrastructure, the area of power supply protection is constantly invaded by various engineering vehicles, especially bulldozers and excavators, which often damage the transmission equipment and pose a serious threat to the security of the power supply. To avoid this kind of external force damage, in the past, the power supply departments would carry out on-site monitoring using video surveillance by attendants and there is no intelligence analysis during this procedure, which increases the fatigue of the monitoring attendants. Meanwhile, the limited human resources cannot meet the needs of the growing scale of power grid monitoring. How to effectively avoid the external force damage caused by engineering vehicles is a challenging problem that researchers urgently need to solve. Recently, several kinds of methods for monitoring power supply areas have been developed that we can use for reference. Surface wave sensors [1] can detect open circuit faults on power cables but are not suitable for early warning of external force damage. Inspection methods based on manned aerial vehicles [2,3] are able to inspect long-distance overhead high-voltage transmission lines within a short time, whereas the disadvantages of this kind of method are equally obvious, which can produce high accident risk and high inspection cost. Another alternative approach to power line inspection is based on unmanned aerial vehicles. Larrauri et al. [4] propose an automatic system to inspect overhead power lines using an unmanned aerial vehicle, which can detect and identify distances and fault location by image processing technology. Luque-Vega et al. [5] propose another unmanned aerial system using the quad rotor helicopter to inspect the devices and components in the high-voltage power line corridors, which use a color camera and a thermal infrared camera. The above methods focus on fault detection and are not very suitable for external force damage detection. Of course, we can also detect engineering vehicles using aerial systems, however, these kinds of methods are often based on the cycle patrol mode, which has difficulty monitoring moving objects in a fixed area in real time.

Another branch of inspection methods is based on visual sensors, which belong to the field of intelligent video surveillance. Surveillance cameras have the characteristics of simple deployment, low cost and easy maintenance. Nowadays, surveillance cameras are also gradually used to monitor the region around the overhead high-voltage power lines. Compared with the artificial inspection method, the intelligent video surveillance method can greatly save human resources, which is adopted increasingly in the field of power line corridors monitoring. It is worth noting that the power supply of surveillance cameras mounted on the electricity tower is a hard problem due to the difficulty of high and low voltage conversion. So, the front-end surveillance cameras have to rely on solar energy, which means we have to reduce the sample rate and capture the low-frequency sample image sequence. The time interval between sequential images is usual 5 min or 10 min, which will affect the selection of monitoring methods. Yang et al. [6] apply binocular cameras to monitor scenes of high-voltage power line corridors and calculate the distance between the camera and the intrusion object. In this method, the background model of scenes is established using Gaussians Mixtures Model (GMM). The disparity between left and right images is established based on Scale Invariant Feature Transform (SIFT) features. This detection method is based on the continuous image sequence, which is not suitable for detecting the object from the low-frequency sample image sequence. Moreover, engineering vehicles invading the monitoring area may remain stationary for a long time, which will lead the objects to blend into the background.

With the introduction of LeNet-5 [7], deep learning especially Convolutional Neural Networks (CNN) methods have been increasingly used in the field of image classification and object detection, which have achieved remarkable results. For object detection, CNN methods can be roughly divided into two categories, one is the two-stage methods based on region proposal and the other is the one-stage methods based on regression. The feature extraction part of these methods is done by the convolutional neural network. Some methods based on region proposal such as R-CNN [8], Fast R-CNN [9] and SPPnet [10], which adopt selective search algorithm to generate candidate boxes. Faster R-CNN [11] uses a region proposal network (RPN) to propose regions, which has promoted the accuracy and the detection speed relative to previous methods of object detection. YOLO [12] and SSD [13] are representative methods which are based on regression. YOLO uses the topmost feature map to predict confidences and bounding boxes for all categories over a fixed grid. SSD detects multiple categories by a single evaluation of the input image. Compared to the detection method based on region proposal, the one-stage methods such as YOLO and SSD have faster processing speed at the expense of accuracy. In our application, detection accuracy is more important relative to the detection speed. Considering all methods of the above, we choose Faster R-CNN as the basic framework. The original detectors are trained on PASCAL VOC [14], which cannot be directly used in our task. So, we modify the network structure and train the detection model in the specific dataset.

For engineering vehicles detection, there are the following challenges: Firstly, the background of power supply scenes is varied. In this case, the detector easily misjudges the background as the object, which can produce unexpected false alarm. Secondly, outdoor lighting changes can also lead to missed detection. In addition, bad weather conditions, such as fog, haze, rain and snow weather can also pose a negative effect on the detection results, which require a more robust detector for our challenging task.

Our main contributions are as follows:

- We designed an intelligent image surveillance system which can automatically detect engineering vehicles that invade into the monitoring area of high-voltage power lines.
- We proposed a modified object detection model based on the framework of Faster R-CNN. We adjusted the position of the ROI (region of interest) pooling layer to make the modified detection model more suitable for the task of engineering vehicles detection.
- We made a scene dataset for power line corridors, which is suitable for training object detection model based on deep learning.

The rest of this paper is organized as follows. Section 2 introduces the overall architecture of the intelligent image surveillance system for monitoring overhead high-voltage power line corridors. Section 3 introduces the structure of the detection model and describes how we modify the structure for our application scenario. Section 4 shows experimental results and analyzes the results to prove the performance of the method proposed. Finally, we make a brief conclusion in Section 5.

## 2. Architecture of the System

**Figure 1:**
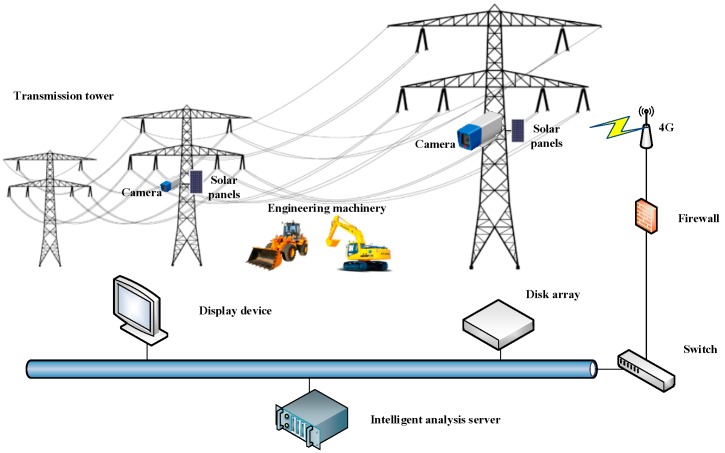
The solution of intelligent surveillance system for engineering vehicles detection, which uses solar energy to supply the monitoring cameras.

The solution of the intelligent surveillance system for power lines consists of network surveillance cameras, solar panels, wireless transmission device, network switch, disk array, display device and intelligent analysis serve, as shown in Figure 1. The cameras rely on 4G signals to send images to the surveillance center. Considering the power supply mode of cameras and the cost of 4G signals transmission, the intelligent video surveillance system can only work during the daytime and the cameras capture a high-definition (HD) picture of scene at intervals which is usually set to be 5 min or 10 min. HD pictures are sent back to the disk array of the surveillance center for storage and are sent to the intelligent analysis server for processing. The network switch is used to connect multiple cameras simultaneously, enabling the system to control multiple monitoring points. Our detection algorithm is embedded into the software module of the intelligent analysis server. Detection results are transmitted to the display device for viewing and are transmitted to the disk array for storage.

**Figure 2:**
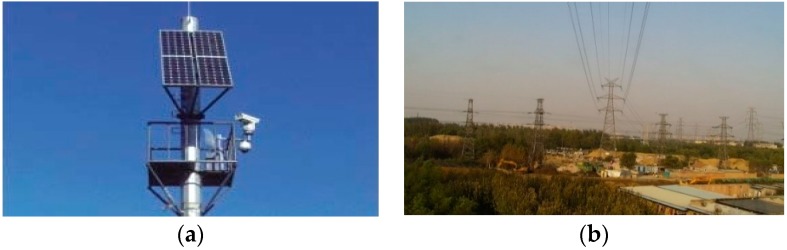
(**a**) The installation position of the camera and the solar panel on the pylon in practical application; (**b**) is the scene of power line corridors captured by the camera.

In the practical application, cameras are installed on the pylon and is 6 m to 10 m above the ground. The camera on each pylon faces the same direction and the monitoring distance of each camera is 300 m. The surveillance center obtains images from the front-end cameras in turn. The actual installation position of the camera and power supply module is as shown in Figure 2a. Figure 2b shows the image taken by camera at one monitoring points.

## 3. The Algorithm Theory

### 3.1. Faster R-CNN

Faster R-CNN is a representative algorithm for object detection based on region proposal. The architecture of Faster R-CNN can be divided into two parts: Region proposal network and detection network. The part of feature extraction is the reduced version of classification model pre-trained on a large-scale hierarchical image database. The classification model mainly consists of convolutional layers and max-pooling layers. In Faster R-CNN, the region proposal networks (RPNs) is used to generate region proposals. RPNs are fully convolutional networks that can be trained end-to-end, which is used to simultaneously conduct regression of region boundaries and scores of objects at each location on a regular grid. The score given by the RPNs evaluates whether the proposed region is an object or a background. The detection network is the same as the Fast R-CNN [9] which shares convolutional features with RPNs. The overall detection process is as follows: The network takes an image of any size as input and uses the convolutional neural network to extract feature maps. The RPNs takes the feature map as input and outputs a set of rectangular object proposals and the score of each proposal. For each region proposal, a fixed-length feature vector is extracted from the feature map by the ROI pooling layer. And each fixed-length feature vector is then fed into a series of fully connected layers. Finally, feature vectors are sent into two parallel output layers, one for generating softmax probabilities for K +  1 classes, the other for generating the coordinates of the corresponding position of bounding boxes.

There are two key technologies in RPN: Anchor mechanism and bounding box regression. In the anchor mechanism, RPNs use a mini network of size n × n to run as a sliding window on the convolutional feature map of the last shared convolution layer. The number of predicted region proposals at each sliding-window location is set to K. Each sliding window is mapped to a low-dimensional feature and after that, the feature is input to two siblings 1×1 convolutional layers: a box-regression layer (reg) and a box-classification layer (cls). The classification layer is implemented as a two class softmax layer to estimate the probability of each proposal. Outputs of the classification layer are 2 K scores (object or background) for K proposals. The regression layer has 4 K outputs that encode the coordinates of K boxes. The anchor is fixed at the center of the sliding window with the corresponding scale and aspect ratio. By default, Faster R-CNN uses 3 scales and 3 aspect ratios to yield K=9 anchors at each sliding window. Bounding box regression is used to adjust the coordinates of the predicted bounding box, so as to obtain a candidate which is closer to the ground-truth box.

### 3.2. Transfer Learning

It is difficult to collect sufficient data that adequately matches the depth of the neural network structure, so researchers usually introduce the idea of transfer learning into the training of convolutional neural networks. Firstly, we pre-train a convolutional neural network on a large-scale database, which is usually used for classification. And then the pre-trained network is performed as an initialization model [15] or a fixed feature extractor [16,17] for the target task. The ILSVRC-2012 challenge dataset is often used by researchers to train deep representations. This dataset contains 1000 object categories from ImageNet [18] and consists of approximately 1.2 million training images, 50,000 validation images and 100,000 test images.

The application of transfer learning in the training process of object detection network is also called finetune. Finetune not only solves the problem of insufficient data but also greatly reduces training time. Moreover, researchers can train deep models with low-configured hardware. Except for the newly defined layer, the weight parameters of all other layers are initialized based on the pre-trained convolutional neural network. Several of the 1000 classes contained in the ImageNet database include bulldozers and other vehicles. Therefore, we can initialize some parameters of the detection model based on the pre-trained model on the ImageNet database. In order to avoid the problem of over-fitting, the network parameters of the previous layers are fixed and the higher part of the network structure is fine-tuned only.

### 3.3. Data

For engineering vehicles that invade into the monitoring area of overhead transmission lines, there is not yet special detection database. We select appropriate pictures from the captured scene images to produce training set and testing set. Bulldozers and excavators are the most common objects that appear at the construction site and are easy to cause short circuit interference to the power lines. Therefore, our purpose is to detect bulldozers and excavators in the monitoring area of overhead transmission lines. The total amount of captured scene images is up to 2.5 terabytes. The data is collected by cameras installed at multiple monitoring points of power line corridors. However, not all images are suitable for training deep object detection model, since most of them are useless data. We remove the images captured at night, the incomplete images, the duplicate images and the images without objects. And finally, we get a total of 2199 annotated images. The total number of objects for the bulldozer category is 968. The total number of objects for the excavator category is 1173.

**Figure 3:**
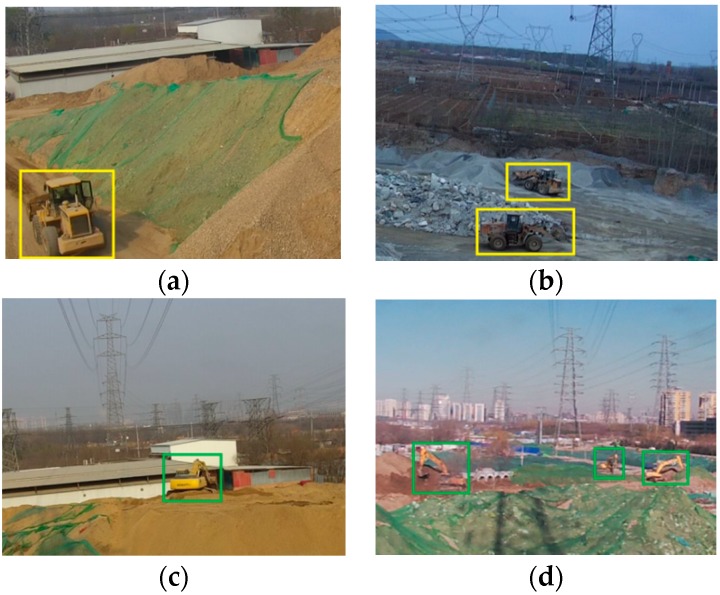
The images of the objects need to be detected in different scenes. The size of the original image is 1280×720 dpi. In order to display the annotated objects well here, we cut the 640×480 dpi size of the area containing the annotated objects in images. (**a**,**b**) show the annotated objects of bulldozer. The color of the annotation box is yellow; (**c**,**d**) show the annotated objects of excavator. The color of the annotation box is green.

Some instances of annotated objects are shown in Figure 3. The method of object labeling used in our database is the same as that of PASCAL VOC database. For excavator and bulldozer classes, the positive examples include the side, the front, the back of the vehicle body and some occlusion situations are also included.

### 3.4. Our Implement

**Figure 4:**
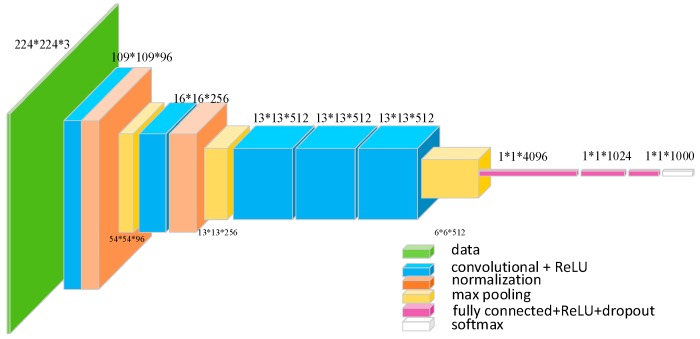
The network architecture of VGG_CNN_M_1024 model. The numbers are shown in form of “size (pixel) * size (pixel) * number,” which specifics the receptive field size and number of convolutional filters.

The entire framework is based on Faster R-CNN. Our task is to detect two types of engineering vehicles that invade into the monitoring area of overhead transmission lines: bulldozers and excavators. Considering that the categories that need to be detected are few and the number of samples in the dataset is small, we choose the pre-trained model of VGG_CNN_M_1024 [19] as the initial weight. Compared to the VGG-16 [20] model which has 13 shareable convolutional layers, the VGG_CNN_M_1024 model has 5 shareable convolutional layers, which is shown in Figure 4.

The first 5 convolutional layers are named in sequence as layer1, layer2, layer3, layer4 and layer5. The kernel size of layer1 is 7 and layer2 is 5. Layer3, layer4 and layer5 have the same kernel size as 3. Meanwhile, Local Response Normalization (LRN) is applied to layer1 and layer2. After the max pooling layer, the size of feature map is reduced to half.

The classification model is trained on the ILSVRC-2012 dataset. The details of the VGG_CNN_M_1024 model can be found in Reference [19].

**Figure 5:**
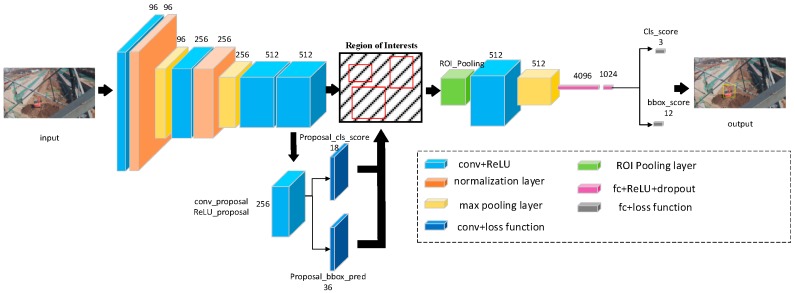
The network architecture of the modified detection model. The entire structure is based on the framework of Faster R-CNN. The numbers in the graph represent the number of the corresponding feature maps. We use the red circle part to represent the position of the predicted bounding boxes. Region proposal network includes a convolutional layer and two sibling fully-connected layers, one is the box-regression layer and the other is the box-classification layer. The text annotation part indicates the type of layer and indicates the number of outputs. Only the new color boxes and the type of layer are explained in the figure.

Faster R-CNN use a multilayer perceptron to classify the extracted object features. In [21], the authors confirmed that a deep region-wise classifier is as important as deep shared features for object detection. They called the region-wise classifier architectures as “Networks on Convolutional feature maps” or “NoCs” for short. Besides, they conducted experiments to prove that convolutional layers are effective for extracting region-wise features and are supplements to the effects of extracting full-image shared features. Inspired by Reference [21], we have modified the network structure of the feature extraction and classification parts. The network architecture of the detection model is shown in Figure 5.

In the task of bulldozer and extractor detection in the monitoring area of overhead transmission lines, there is no need to extract deeper features because the size of object is not very large. At the same time, because of the complex background and various shapes of objects, it is necessary to enhance the recognition ability of the classifier. In addition to the deep feature extractor, the depth of the classifier is also important for the accuracy of object detection, which is ignored by the original Faster R-CNN. The convolution layer enables the classifier to learn the region-wise features which are suitable for the region of interest. The region-wise features are complemented with the shared features of the full image to improve the accuracy of the object detection model. We set the layer4 and its previous layers as the feature extraction network. The layer5 and the first 2 fully connected layers are applied as initial weights for the object classification network. This method adds one convolutional layer to the object classification network in order to improve the classification performance of the network. The shallow convolutional layers extract high-resolution features of the input image. These features usually contain common information such as colors, edges, textures and so on. With the deepening of the network layer, the degree of non-linearity increases and the features extracted by the convolutional layer become more semantic. Considering these characteristics, we freeze the weights of the layer1 The parameters of other convolutional layers and the first 2 fully connected layers are fine-tuned based on the weights of the pre-trained VGG_CNN_M_1024 model.

The training process is the same as that of Reference [11]. First, we normalized the images which are input into the convolutional layers. The minimum side of the image is normalized to be 600 pixels. If the minimum side of the image is less than 600 pixels, we normalize the longer side to be 1000 pixels and the aspect ratio is preserved. In RPNs, we use 9 scales of anchor boxes, the size of which are related to the normalized images. The 3 areas of the anchor box are $128^{2}$, $256^{2}$ and $512^{2}$ pixels, respectively corresponding to the 3 aspect ratios. The aspect ratios of anchor are 1:1, 1:2 and 2:1.

In the step of generating region proposal, we use the 3×3 window to slide on feature maps. We assume that the size of feature maps extracted by convolutional layers is W×H. Because the anchor is located at the center of the sliding window and each anchor generates 9 candidate regions, W×H×9 candidate regions can be obtained on the final feature map. In the training process, we ignore all the anchors which pass through the edges of the image. And for the highly overlapping proposals, we eliminate overlapping proposals by using non-maximal suppression (NMS) method. These operations help to reduce redundancy and avoid introducing error items, so as to ensure the effectiveness of training.

## 4. Results and Discussion

By modifying the network structure of the feature extraction and classification, the detection model is more suitable for detecting engineering vehicles which invade into the monitoring area of overhead transmission lines. We performed experiments based on Caffe [22] to test the robustness of the detection model. We used a graphics workstation to run our experiments with an Intel Xeon with 3.5 GHz, 64 GB RAM, NVDIA TESLA K40 and a Windows 7 (64 bits) operating system.

### 4.1. Experimental Evaluation Criteria

There are many evaluation criteria in the field of object detection but the meanings of different evaluation criteria are often confused. The brief introduction of several commonly used evaluation criteria are as follows.

Firstly, there are 4 basic definitions:

True Positive (TP): which means an instance belongs to positive class and is also determined to be a positive one.

False Negative (FN): which means an instance belongs to positive class but is determined to be a negative one. This indicates that the detection model fails to detect the instance.

False Positive (FP): which means an instance belongs to negative class but is determined to be a positive one. This indicates that the detection model has misjudged the instance.

True Negative (TN): which means an instance belongs to negative class and is also determined to be a negative one.

According to the definition above, the calculation formulas of the common evaluation criteria are given below:

The precision rate (P) reflects the proportion of true positive examples in the positive case determined by the detector.

(1) $$P=\frac{\mathrm{TP}}{\mathrm{TP}+\mathrm{FP}},$$

The recall rate (R) reflects the proportion of positive cases which are judged correctly. RR is also called True Positive Rate.

(2) $$R=\frac{\mathrm{TP}}{\mathrm{TP}+\mathrm{FN}},$$

False Negative Rate (FNR) reflects the proportion of positive cases being missed, which is also called Missing Alarm.

(3) $$\mathrm{FNR}=\frac{\mathrm{FN}}{\mathrm{TP}+\mathrm{FN}},$$

False Alarm (FA) reflects the proportion of negative cases which are judged to be positive examples.

(4) $$\mathrm{FA}=\frac{\mathrm{FP}}{\mathrm{TP}+\mathrm{FP}},$$

The Average Precision (AP) is the measure of the performance of the classifier. An important criterion commonly used to evaluate an object detection algorithm is whether the detector correctly predicts the category of the object in the box. The quantitative measure of this standard is the mean Average Precision (mAP). The value of mAP is obtained by averaging the Average Precision of all the categories.

(5) $$\mathrm{AP}=\int_{0}^{1}P(R)dR,$$

### 4.2. Experimental Results and Analysis

In order to overcome the effects of overfitting, we use a 5-fold cross-validation method to evaluate the detection performance of the model. We divide dataset D into 5 mutually exclusive subsets of similar size, that is

(6) $$\;D=D_{1}\cup D_{2}\cup D_{3}\cup D_{4}\cup D_{5},D_{i}\cap D_{j}=\emptyset (i\neq j),$$

Each subset maintains consistency in data distribution as much as possible. During the training process, the union of 4 subsets is used as the training set at a time and the remaining subset is used as the test set. After 5 times of training and testing, we obtained the results of each test and the weighted average value of results. The experimental dataset contains 2199 images we selected from the scene images and their corresponding annotation information. In the 5-fold cross-validation experiments, the number of the objects included in the training set and the testing set for each experiment is shown in the Table 1.

The number of the images in the training set of experiment-2 is 1760 and the number of test images is 439. In the other experiments, the number of the images in the training set and the testing set are 1759 and 440 respectively. Since the number of objects contained in each image is different, the number of objects in each experiment is different, which is shown in Table 1. In each experiment, the training set contains 774 bulldozers and 938 excavators on average and the testing set contains 193 bulldozers and 235 excavators on average.

Table 2 shows the average precision (AP) values of bulldozers and excavators. The last line in the table shows the mean average precision (mAP) value on the testing set of each experiment. We compared the experimental results of the Faster R-CNN baseline and our modified detection model. We calculated the average value of the results of 5-fold cross-validation experiments. Both networks use the pre-trained VGG_CNN_1024_M classification model. For Faster R-CNN baseline, the AP value of bulldozers is 89.22% and that of excavators is 89.02%; the mAP value for 5-fold cross-validation experiments is 89.12%. For our modified detection model, the AP value of bulldozers is 89.77% and that of excavators is 90.08%; the mAP value for 5-fold cross-validation experiments is 89.93%. From the results, we can see that our modified model based on the framework of Faster R-CNN indeed improve the detection accuracy.

From Table 1 and Table 2, we can see that the number of the samples in training set has some influence on the detection performance of the model. In the testing process, the model gives the coordinate information of the predicted object box and the probability value of the category. According to the coordinate information provided by the detection model, the system labels the predicted object on the original image with a color box, which is also used as a way to alarm. If the probability value of the category is less than the detection threshold, the system will not display the predicted box. In the 5-fold cross-validation experiments, we set the detection threshold of our modified detection model to 0.85.

The detection results of bulldozers are shown in Table 3 and the detection results of excavators are shown in Table 4. The evaluation criteria for the detection results are given in Section 4.1. These criteria are often used to measure the performance of the detection system in industrial applications. Because there are more samples for the excavator, the overall detection performance for the excavator is better.

**Figure 6:**
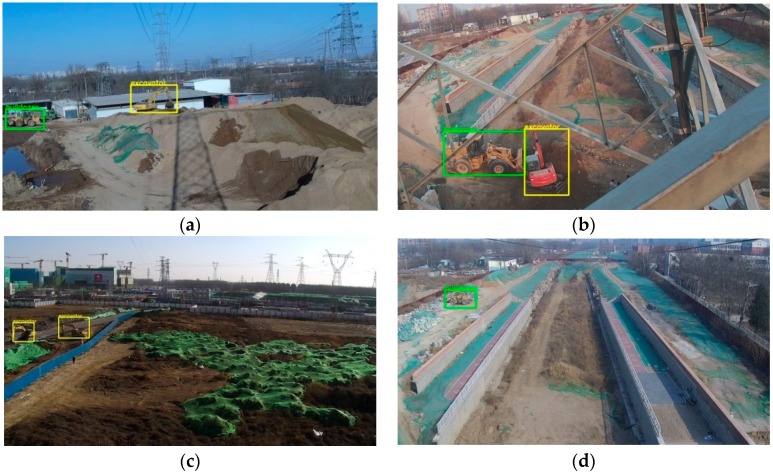
The detection results of varied scenes. The bulldozer in the picture is labeled in green and the excavator is labeled in yellow. (**a**) The whole image is 62 times larger than object areas; (**b**) the whole image is about 27 times larger than object areas; (**c**) the whole image is about 56 times larger than object areas; (**d**) The whole image is about 149 times larger than the object area.

The detection time of the modified model for each image is about 0.32 s and the detection time of original Faster R-CNN is similar to our improved method. Some detection results are shown as Figure 6. As can be seen from Figure 6, the detection model can accurately locate and identify bulldozers and excavators in the background of complex and diverse circumstances, which shows that the detection model can effectively extract the feature information of objects and can distinguish it from the feature information of the complex background.

**Figure 7:**
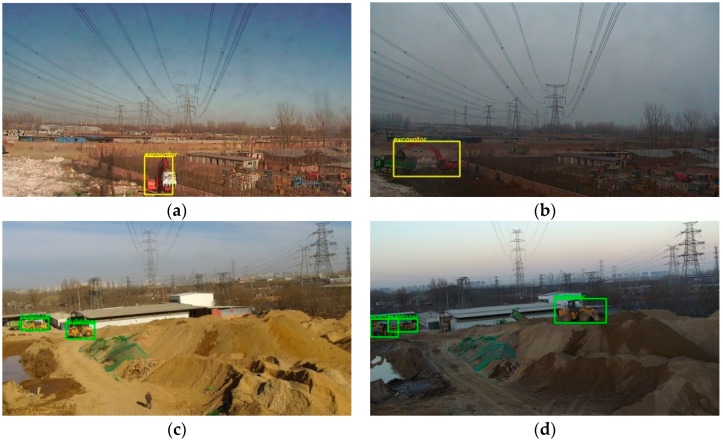
The detection results under different illumination conditions in the same scene. (**a**) The detection results of excavator under good illumination condition. The whole image is about 57 times larger than the object area; (**b**) the detection result of excavator under dim lighting condition. The whole image is about 30 times larger than the object area; (**c**) the detection result of bulldozer under good illumination condition. The whole image is about 159 times larger than object areas; (**d**) the detection result of bulldozer under dim lighting condition. The whole image is about 56 times larger than object areas.

As mentioned in the second section, lighting changes are also one of the challenges we need to face in the task. The entire detection system is required to have good performance during the daytime. As shown in Figure 7, our detection system overcomes the adverse effects of light on the detection results to some extent.

**Figure 8:**
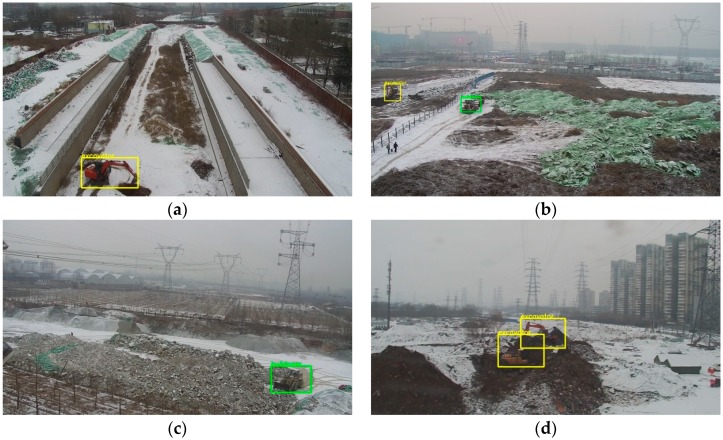
The detection results of different scenes on snow days. (**a**) There is an excavator in the image and the whole image is about 40 times larger than the object area; (**b**) There is an excavator and a bulldozer in the image. The image is about 256 times larger than objects area; (**c**) There is a bulldozer in the image. The whole image is about 67 times larger than the object area; (**d**) there are 2 excavators. The whole image is about 50 times larger than objects area.

We also tested the robustness of the detection system under severe weather conditions. Severe weather conditions include rain, snow and fog. There are few images collected in severe weather in the dataset. However, this kind of situation cannot be ignored in practical application. Figure 8 shows the detection results of different scenes on snow days. The snow on the ground changes the color characteristics of the background, which is equivalent to adding noise to the image. Observing the results, the model can avoid the influence of the negative factors on our test dataset.

**Figure 9:**
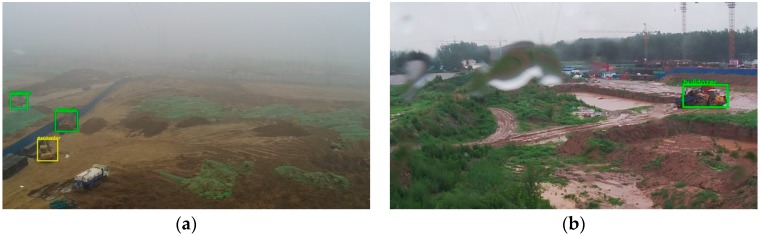
The detection results under raining and fogging weather. (**a**) There is an excavator and 2 bulldozers. The whole image is about 180 times larger than object areas; (**b**) there is a bulldozer in the image. The image is about 96 times larger than the object area.

As shown in Figure 9a, the image captured by the camera in foggy weather is blurred. Some detailed features of the object are not obvious enough in this case. In the scene images collected on the rainy day, as shown in Figure 9b, rain falling on the lens blurs the camera’s view and the light is usually dimmer in this weather. These factors increase the difficulty of the testing. From the test results, we can see that our detection system is robust to severe weather. In most cases, the system can detect engineering vehicles invading the monitoring area of overhead high-voltage transmission lines.

**Figure 10:**
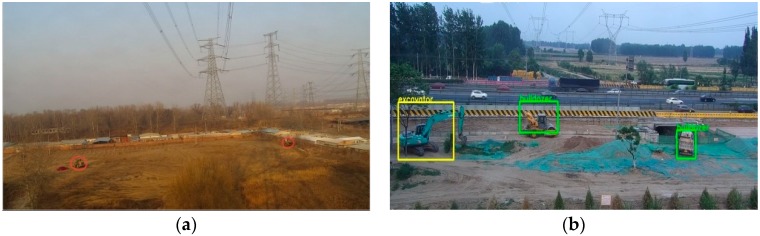
(**a**) The object labeled with a red circle is a bulldozer. The bulldozer on the left is bigger. The original image size is 527 times larger than the left bulldozer and is 732 times larger than the right bulldozer; (**b**) The truck located on the far right of the image has been wrongly detected as a bulldozer.

There are false detections and missed detections in the results. The system will make false alarm behavior due to the false detection results and missed detection will make the system fail to give an alarm in time when the engineering vehicle invades the monitoring area. As shown in Figure 10a, bulldozers labeled in the red circle have not been detected by the system. This is due to the fact that the sizes of positive samples in the training set are much larger. The original image in the training set is about 250 times larger than the objects. The size of these 2 bulldozers is much smaller than that of the positive sample in the training set. Without training with the small object samples, the detection performance of the detection system for this situation is not satisfactory. There is another situation in which the background is mistakenly detected as the object in Figure 10b. This may be due to the fact that the rear of the truck is similar to the back of the bulldozer, which is very hard to distinguish in the case of small size.

## 5. Conclusions

We proposed a novel intelligent surveillance system for detecting engineering vehicles that invade the monitoring areas of overhead high-voltage transmission lines. The detection model is based on the framework of Faster R-CNN. In order to achieve good performance in our detection task, we modified the structure of the detection model. The experimental results presented in Section 4 proved that our method is robust to adverse factors such as light variation, severe weather and so on. We fine-tuned the modified model to make up for the shortage of data but it is undeniable that increasing the amount of data can further enhance the performance of the object detection model. In future work, we will focus on the more appropriate network architecture designed for engineering vehicles detection, so that we can handle more complex application scenes.

## Figures and Tables

**Table 1:** The number of objects in each experiment.

Dataset	Category	Experiment 1	Experiment 2	Experiment 3	Experiment 4	Experiment 5
training set (4-fold)	bulldozer	767	769	763	797	776
	excavator	767	769	763	797	776
testing set (1-fold)	bulldozer	201	199	205	171	192
	excavator	245	245	229	241	213

**Table 2:** Test results.

	Category	Experiment 1	Experiment 2	Experiment 3	Experiment 4	Experiment 5	Average
Bulldozers AP (%)	Faster R-CNN	89.23	89.48	88.98	88.89	89.50	89.22
	ours	89.34	89.87	90.30	89.20	90.07	89.77
Excavator AP (%)	Faster R-CNN	89.80	89.66	88.83	89.57	86.95	89.02
	ours	90.03	90.62	89.63	90.01	90.10	90.08
mAP (%)	Faster R-CNN	89.52	89.57	88.91	89.23	88.23	89.12
	ours	89.69	90.25	89.97	89.61	90.09	89.93

**Table 3:** Detection results of bulldozers.

Evaluation Criteria	Experiment 1	Experiment 2	Experiment 3	Experiment 4	Experiment 5	Average
P (%)	97.98	97.54	97.41	98.77	97.77	97.87
R (%)	98.98	98.51	98.43	98.77	98.87	98.71
FNR (%)	1.02	1.49	1.57	1.23	1.13	1.29
FA (%)	2.02	2.46	2.59	1.23	2.23	2.13

**Table 4:** Detection results of excavators.

Evaluation Criteria	Experiment 1	Experiment 2	Experiment 3	Experiment 4	Experiment 5	Average
P (%)	98.19	97.85	97.10	98.12	98.91	98.02
R (%)	98.19	98.28	98.53	99.05	98.38	98.49
FNR (%)	1.81	1.72	1.47	0.95	1.62	1.51
FA (%)	1.81	2.15	2.90	1.88	1.09	1.98

## References

[1] Alam M.N., Bhuiyan R.H., Dougal R.A., Ali M. (2013). Design and application of surface wave sensors for nonintrusive power line fault detection. IEEE Sens. J..

[2] Jones D.I., Earp G.K. (2001). Camera sightline pointing requirements for aerial inspection of overhead power lines. Electr. Power Syst. Res..

[3] Golightly I., Jones D. (2003). Corner detection and matching for visual tracking during power line inspection. Image Vis. Comput..

[4] Larrauri J.I., Sorrosal G., González M. Automatic system for overhead power line inspection using an unmanned aerial vehicle—RELIFO project. Proceedings of the 2013 International Conference on Unmanned Aircraft Systems (ICUAS).

[5] Luque-Vega L.F., Castillo-Toledo B., Loukianov A., Gonzalez-Jimenez L.E. Power line inspection via an unmanned aerial system based on the quadrotor helicopter. Proceedings of the MELECON 2014—2014 17th IEEE Mediterranean Electrotechnical Conference.

[6] Fu Y., Rong S.A., Zhao W.B., Shen H. Research on monitoring device for indicating external damage risk of overhead line based on image recognition technology with binocular vision cameras. Proceedings of the International Conference on Condition Monitoring and Diagnosis.

[7] Lécun Y., Bottou L., Bengio Y., Haffner P. (1998). Gradient-based learning applied to document recognition. Proc. IEEE.

[8] Girshick R., Donahue J., Darrell T., Malik J. (2016). Region-based convolutional networks for accurate object detection and segmentation. IEEE Trans. Pattern Anal. Mach. Intell..

[9] Girshick R. Fast R-CNN. Proceedings of the IEEE International Conference on Computer Vision (ICCV).

[10] He K., Zhang X., Ren S., Sun J. (2015). Spatial pyramid pooling in deep convolutional networks for visual recognition. IEEE Trans. Pattern Anal. Mach. Intell..

[11] Ren S., He K., Girshick R., Sun J. (2017). Faster R-CNN: Towards real-time object detection with region proposal networks. IEEE Trans. Pattern Anal. Mach. Intell..

[12] Redmon J., Divvala S., Girshick R., Farhadi A. You only look once: Unified, real-time object detection. Proceedings of the 2016 IEEE Conference on Computer Vision and Pattern Recognition (CVPR).

[13] Liu W., Anguelov D., Erhan D., Szegedy C., Reed S. SSD: Single shot multibox detector. Proceedings of the European Conference on Computer Vision.

[14] Everingham M., Gool L.V., Williams C.K.I., Winn J., Zisserman A. (2010). The pascal visual object classes (voc) challenge. Int. J. Comput. Vis..

[15] Donahue J., Jia Y., Vinyals O., Hoffman J., Zhang N., Tzeng E., Darrell T. (2014). Decaf: A deep convolutional activation feature for generic visual recognition. Proceedings of the 31st International Conference on Machine Learning, ICML 2014.

[16] Zeiler M.D., Fergus R. (2014). Visualizing and understanding convolutional networks. Proceedings of the 13th European Conference on Computer Vision, ECCV 2014.

[17] Shin H.C., Roth H.R., Gao M., Lu L., Xu Z., Nogues I., Yao J., Mollura D., Summers R.M. (2016). Deep convolutional neural networks for computer-aided detection: CNN architectures, dataset characteristics and transfer learning. IEEE Trans. Med. Imaging.

[18] Russakovsky O., Deng J., Su H., Krause J., Satheesh S., Ma S., Huang Z., Karpathy A., Khosla A., Bernstein M. (2015). Imagenet large scale visual recognition challenge. Int. J. Comput. Vis..

[19] Chatfield K., Simonyan K., Vedaldi A., Zisserman A. (2014). Return of the devil in the details: Delving deep into convolutional nets. arXiv.

[20] Simonyan K., Zisserman A. (2014). Very deep convolutional networks for large-scale image recognition. arXiv.

[21] Ren S., He K., Girshick R., Zhang X., Sun J. (2015). Object detection networks on convolutional feature maps. IEEE Trans. Pattern Anal. Mach. Intell..

[22] Jia Y., Shelhamer E., Donahue J., Karayev S., Long J. Caffe: Convolutional architecture for fast feature embedding. Proceedings of the 22nd ACM International Conference on Multimedia.

